# Protective Immunity against *Chlamydia psittaci* Lung Infection Induced by a DNA Plasmid Vaccine Carrying *CPSIT_p7* Gene Inhibits Dissemination in BALB/c Mice

**DOI:** 10.3390/ijms24087013

**Published:** 2023-04-10

**Authors:** Chuan Wang, Yingqi Jin, Jiewen Wang, Kang Zheng, Aihua Lei, Chunxue Lu, Shuzhi Wang, Yimou Wu

**Affiliations:** 1 Institute of Pathogenic Biology, School of Basic Medicine, Hengyang Medical College, University of South China, Hengyang 421001, China; 2 Hunan Provincial Key Laboratory for Special Pathogens Prevention and Control, University of South China, Hengyang 421001, China; 3 Department of Clinical Laboratory, Hengyang Central Hospital, Hengyang 421001, China; 4 Department of Pharmacology, School of Pharmaceutical Science, Hengyang Medical College, University of South China, Hengyang 421001, China

**Keywords:** *Chlamydia psittaci*, CPSIT_p7, DNA vaccine, dissemination, protective immunity

## Abstract

*Chlamydia psittaci* (*C. psittaci*), a zoonotic pathogen, poses a potential threat to public health security and the development of animal husbandry. Vaccine-based preventative measures for infectious diseases have a promising landscape. DNA vaccines, with many advantages, have become one of the dominant candidate strategies in preventing and controlling the chlamydial infection. Our previous study showed that CPSIT_p7 protein is an effective candidate for a vaccine against *C. psittaci*. Thus, this study evaluated the protective immunity of pcDNA3.1(+)/CPSIT_p7 against *C. psittaci* infection in BALB/c mice. We found that pcDNA3.1(+)/CPSIT_p7 can induce strong humoral and cellular immune responses. The IFN-γ and IL-6 levels in the infected lungs of mice immunized with pcDNA3.1(+)/CPSIT_p7 reduced substantially. In addition, the pcDNA3.1(+)/CPSIT_p7 vaccine diminished pulmonary pathological lesions and reduced the *C. psittaci* load in the lungs of infected mice. It is worth noting that pcDNA3.1(+)/CPSIT_p7 suppressed *C. psittaci* dissemination in BALB/c mice. In a word, these results demonstrate that the pcDNA3.1(+)/CPSIT_p7 DNA vaccine has good immunogenicity and immunity protection effectiveness against *C. psittaci* infection in BALB/c mice, especially pulmonary infection, and provides essential practical experience and insights for the development of a DNA vaccine against chlamydial infection.

## 1. Introduction

The diseases caused by *Chlamydia* are widely prevalent in animals and humans. Under certain circumstances, *Chlamydia* can transmit to animals or humans. In addition, cross-infection between different hosts can also be achieved, causing severe harm to human public health, agriculture, and animal husbandry [1,2]. An active and effective vaccine is the optimal program to prevent the widely spread chlamydial diseases [3]. The *Chlamydia* vaccine has been in research for hundreds of years. The development process has significantly progressed from the original whole-bacteria vaccine to the subunit vaccine concentrated in recent decades [4,5]. As an emerging chlamydial vaccine in the past two decades, the DNA vaccine enjoys its advantages of safety and convenience, and can stimulate antigen expression in vivo [6].

The nucleic acid sequences of exogenous vaccine antigens, currently mainly selected subunit vaccine antigens, were inserted into eukaryotic expression vector plasmids containing strong eukaryotic cell promoters to induce expression of the corresponding antigens [6,7]. The research on chlamydial DNA vaccines started in 1997 [8], and primarily targeted the genes *ompA*, *Pgp3*, and *HSP60* [9,10,11,12,13]. Zhang et al. constructed the MOMP/pcDNA3.1 eukaryotic expression plasmid. They found that the DNA-MOMP vaccine could promote the subunit antigen-immunized mice to produce more robust protection against *Chlamydia trachomatis* (*C. trachomatis*) infection [14]. The above finding also provides a new idea for developing a chlamydial DNA vaccine in the future.

*Chlamydia* vaccines based on a chlamydial plasmid also experienced the development trend of available vaccines, mainly including live attenuated vaccines, subunit recombinant vaccines, and DNA vaccines [15]. The plasmid-deficient strain is usually used as a live attenuated vaccine because of its low toxicity as a subunit vaccine with good immunogenicity [16]. Plasmid proteins Pgp3 and Pgp4 have been widely reported in anti-infection experiments of *C. trachomatis* and *Chlamydia muridarum* (*C. muridarum*) [17]. Our previous studies have proven that the *C. psittaci* plasmid proteins CPSIT_p7 and CPSIT_p8 possess desirable immunogenicity as well. The CPSIT_p7 polypeptide vaccine antigen was further constructed, and animal experiments showed good anti-infection protection [18,19]. Donati et al. [20] constructed a pCMV-KA-Pgp3 eukaryotic vector, and then immunized C3H/HeN mice to evaluate the protective effect against *C. trachomatis* vaginal infection. The DNA vaccine inhibited *C. trachomatis* infection and the transmission of *Chlamydia* from the lower to the upper genital tract. Mice inoculated with an empty carrier alone did not have a similar protective effect. All DNA-vaccinated mice remained resistant to *Chlamydia* reinfection. Li et al. [21] also found that the DNA vaccine targeting the *Pgp3* gene could also inhibit the ascending infection of *C. trachomatis* in mice after immunization. A pORF5 DNA vaccine was used to evaluate the protective effect against *C. trachomatis* reproductive tract infection in mice. The results showed that DNA vaccination significantly reduced *Chlamydia* load and accelerated the clearance of infection in mice. However, there needs to be more reports on the *C. psittaci* Pgp3 DNA vaccine.

Pgp3 is one of the virulence factors of *Chlamydia*, which has good immunogenicity and is an excellent immune antigen. Good immune protection has been confirmed in animal experiments [17,22]. The autoimmunity of plasmid protein Pgp3 and its role in the pathogenesis of *Chlamydia* determine that a protein DNA vaccine can also be used as a candidate antigen for antichlamydial infection vaccine research. Based on the previous studies, we utilized Pgp3 to design a DNA vaccine for *C. psittaci*.

In this study, pcDNA3.1(+)/CPSIT_p7 was first constructed. The immunogenicity and immune-protective effects of intramuscular injection with pcDNA3.1(+)/CPSIT_p7 against *C. psittaci* intranasal infection in BALB/c mice were evaluated. Therefore, we further evaluated whether DNA vaccines could inhibit the ability of *C. psittaci* to spread to multiple tissues and organs. This study will provide an experimental basis for preventing and treating the anti-*C. psittaci* infection DNA vaccine.

## 2. Results

### 2.1. Plasmid pcDNA3.1(+)/CPSIT_p7 Was Successfully Constructed and Expressed CPSIT_p7 Protein in HeLa Cells

**Figure 1:**
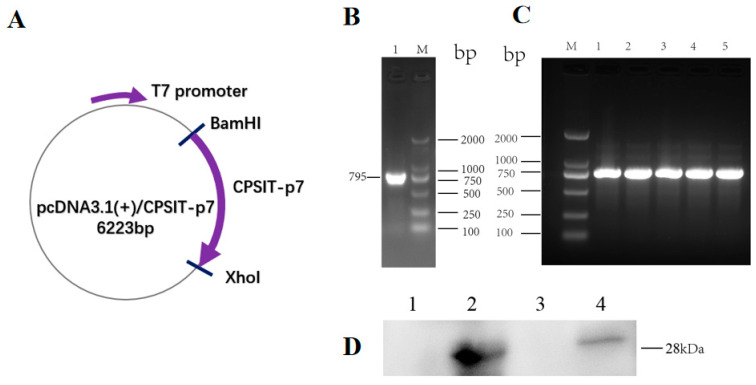
Diagram showing pcDNA3.1(+)/CPSIT_p7 plasmid structure and identification of the construction and function via PCR amplification and Western blot. (**A**) The structure diagram of pcDNA3.1(+)/CPSIT_p7 plasmid. (**B**) PCR amplification of *CPSIT_p7* gene. The *CPSIT_p7* gene (795bp) and the 2000 bp DNA ladder were visualized in lanes one and M, respectively. (**C**) Bacteria PCR amplification of pcDNA3.1(+)/CPSIT_p7. The 2000 bp DNA ladder was loaded in lane M, and the pcDNA3.1(+)/CPSIT_p7 DNA fragments (795bp) were seen in lanes one to five. (**D**) Western blot analysis showing the expression of CPSIT_p7 protein in HeLa cells. Plasmid pcDNA3.1(+)/CPSIT_p7 was transfected into HeLa cells using lipofectamine 3000. After 48 h, these cells were lysed and immunoreacted with a rabbit anti-*C. psittaci* antibody. Lane one, untransfected HeLa cells (PBS); lane two, recombinant protein CPSIT_p7; lane three, HeLa cells transfected with empty pcDNA3.1(+) vector; lane four, HeLa cells transfected with pcDNA3.1(+)/CPSIT_p7 plasmid.

Briefly, the construction method of pcDNA3.1(+)/CPSIT_p7 plasmid was that the target *CPSIT_p7* gene (795bp) was inserted into empty pcDNA3.1(+) vector, which was digested with the restriction enzymes *BamHI* and *XhoI* (Figure 1A). With a pair of corresponding specific primers, *C. psittaci* 6BC genomic DNA and DNA extracted from *E. coli* JM 109, which has pcDNA3.1(+)/CPSIT_p7 plasmid as template, respectively, we used PCR to amplify the target gene. The *CPSIT_p7* gene (795bp) was found in 1.5% agarose gel electrophoresis of PCR amplification products (Figure 1B,C). In order to assess the function of pcDNA3.1(+)/CPSIT_p7 plasmid, we identified the expression of CPSIT_p7 protein in HeLa cells by Western blot with rabbit anti-*C. psittaci* antibody as the primary antibody, and HRP-labeled goat anti-rabbit IgG as the secondary antibody. The results showed that there was an apparent protein of 28 kDa both in the plasmid pcDNA3.1(+)/CPSIT_p7 group and the recombinant protein CPSIT_p7 group, while the empty pcDNA3.1(+) vector group and PBS group had no hybrid zone in the corresponding position (Figure 1D). Western blot results confirmed that the plasmid pcDNA3.1(+)/CPSIT_p7 could express CPSIT_p7 protein in eukaryotic cells.

### 2.2. pcDNA3.1(+)/CPSIT_p7 Vaccine Induced Strong Antibody-Specific Responses in BALB/c Mice

**Figure 2:**
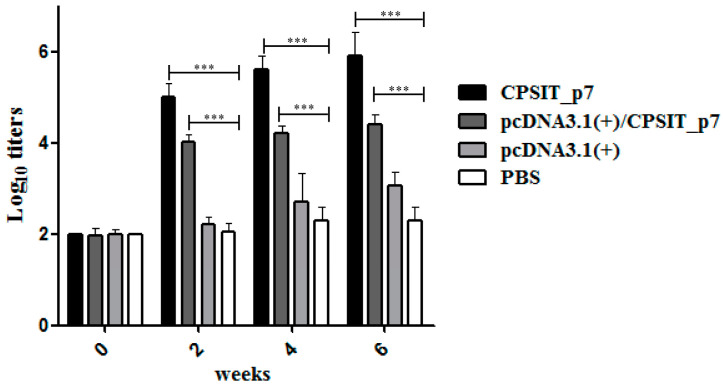
ELISA showing the antibody levels in the mice immunized with different vaccines. Mice were intramuscularly immunized three times at 2-week intervals with 100 μg of the pcDNA3.1(+)/CPSIT_p7 plasmid, 100 μg of pcDNA3.1(+) vector, 30 μg of recombinant CPSIT_p7 protein, or 100 μL PBS, respectively. Serum samples were collected, and the levels of serum IgG were analyzed by ELISA. The means and standard derivations for the antibody levels were calculated from five mice. *** *p* < 0.001.

Using ELISA assay, we detected the titers of specific IgG antibody against recombinant CPSIT_p7 protein induced at different time points after immunization, respectively, with pcDNA3.1(+)/CPSIT_p7, pcDNA3.1(+) vector, recombinant CPSIT_p7 protein, or PBS. Figure 2 shows that specific IgG antibody levels of pcDNA3.1(+)/CPSIT_p7 group mice increased significantly in the second week. The peaking titers appeared on the sixth week, while recombinant CPSIT_p7 protein group mice induced higher titers level. On the contrary, little specific IgG antibody could be found in the pcDNA3.1(+) vector and PBS group.

### 2.3. Vaccinations with pcDNA3.1(+)/CPSIT_p7 Plasmid Effectively Decreased the C. psittaci load in the Lungs of BALB/c Mice

**Figure 3:**
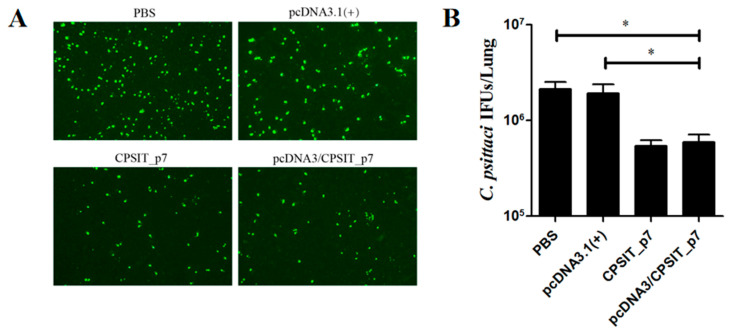
The *C. psittaci* load in the lungs of mice injected with a different vaccine after the *C. psittaci* challenge. The supernatants of lung homogenates of immunized mice were collected 10 days after being challenged intranasally with 5 × 10^5^ IFUs of *C. psittaci*. (**A**) The immunofluorescent images of HeLa cells incubated with the supernatants of lung homogenates. In order to visualize the *C. psittaci* inclusions, samples were stained with rabbit anti-*C. psittaci* sera (green). (**B**) The number of inclusions expressed as IFUs. Each bar indicates the mean ± standard deviation of the *C. psittaci* titers (IFU/lung) in the lung homogenates from six mice per group in three independent data points. * *p* < 0.05.

In order to evaluate the immune protective efficacy of the vaccine, the supernatants of lung homogenates of all mice were collected on day 10 after being challenged intranasally with 5 × 10^5^ IFUs of *C. psittaci.* The *C. psittaci* load was determined by IFA. Inclusion was observed by fluorescence microscopy assay (Figure 3A), and the number of inclusions was counted based on the area and dilutions, and expressed as IFUs (Figure 3B). The number of inclusions in the lungs of pcDNA3.1(+)/CPSIT_p7 plasmid group ((5.93 ± 2.05) × 10^5^ IFUs) as well as CPSIT_p7 protein group ((5.43 ± 1.35) × 10^5^ IFUs) were significantly lower than that of pcDNA3.1(+) vector group ((19.30 ± 7.89) × 10^5^ IFUs) and PBS group ((21.40 ± 6.89) × 10^5^ IFUs). However, there was no obvious difference between the pcDNA3.1(+)/CPSIT_p7 plasmid group and the CPSIT_p7 protein group.

### 2.4. pcDNA3.1(+)/CPSIT_p7 Vaccine Regulated the Production of IL-6 and IFN-γ in BALB/c Mice

**Figure 4:**
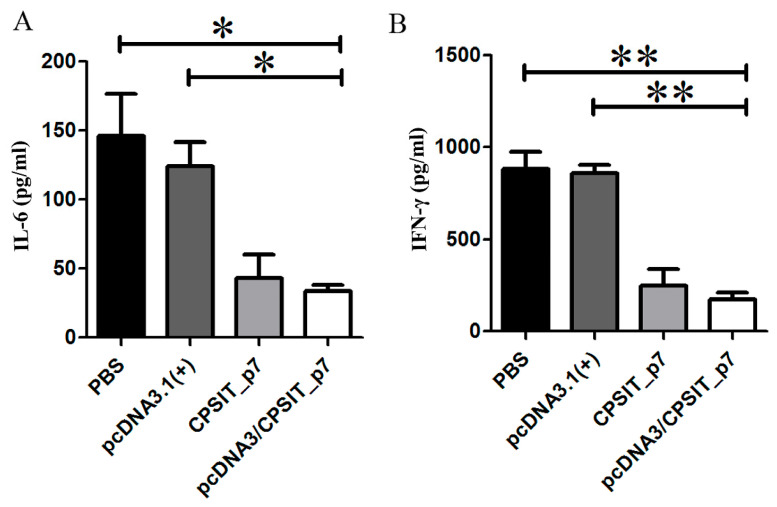
Cytokine levels in the lungs from *C. psittaci*-infected mice of each group. The supernatants of lung homogenates were collected 10 days after being challenged intranasally with 5 × 10^5^ IFUs of *C. psittaci*. The production of IL-6 (**A**) and IFN-γ (**B**) in lung supernatants was detected by ELISA. Values shown are mean ± standard deviation from five mouse lungs in three independent experiments. * *p* < 0.05. ** *p* < 0.01.

Various cytokines are essential in regulating host immune and inflammatory responses during *C. psittaci* infection. To evaluate cytokine-related vaccination regulation, we collected the supernatants of lung homogenates 10 days after being challenged intranasally, to measure the levels of IL-6 and IFN-γ by ELISA. As shown in Figure 4A,B, the content of IL-6 and IFN-γ in the lungs of pcDNA3.1(+)/CPSIT_p7 plasmid group and CPSIT_p7 protein group were both significantly lower than that in the pcDNA3.1(+) vector group and PBS group. However, no significant difference was observed between pcDNA3.1(+)/CPSIT_p7 plasmid and CPSIT_p7 protein groups.

### 2.5. pcDNA3.1(+)/CPSIT_p7 Vaccine Diminished Pulmonary Pathological Lesions and Reduced the C. psittaci Load in the Lung of the Infected BALB/c Mice

**Figure 5:**
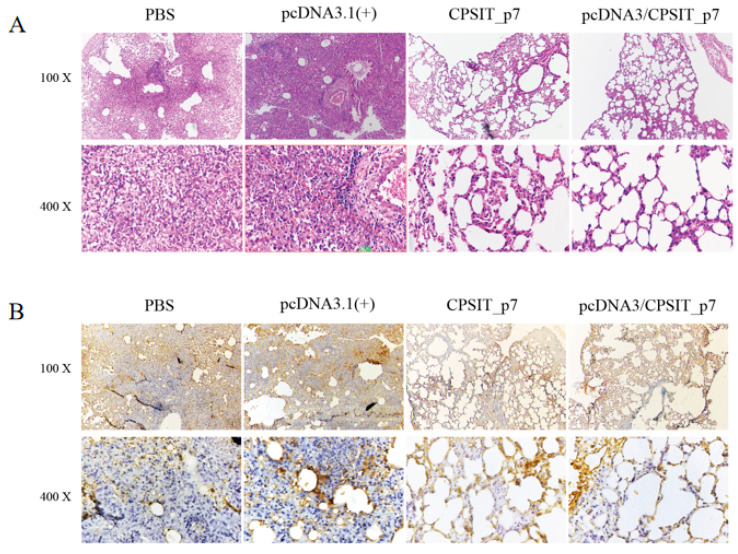
Inflammatory pathologies and S–P immunohistochemistry of *C. psittaci*-infected lung tissue. (**A**) Representative results of hematoxylin–eosin (H&E) staining in the lungs of control and immunized mice 10 days after infection with *C. psittaci*. (**B**) Illustrative immunohistochemical photographs of sections from different groups of infected mice. The Streptavidin–Peroxidase (S–P) immunohistochemistry kit with a rabbit anti-*C. psittaci* 6BC, as the first antibody, was used to detect *C. psittaci* inclusion. Brown granules represent the infiltration of *C. psittaci* inclusions.

*C. psittaci* primarily causes respiratory system disease and inflammatory pathologies. S–P immunohistochemistry in the lung tissue on day 10 after infection was observed by H&E and IHC analyses for a further evaluation of the immune protective efficacy of the vaccines. The results showed that both pcDNA3.1(+)/CPSIT_p7 plasmid group and CPSIT_p7 protein group of mice maintained relative integrity of pulmonary tissue structure, though some alveolar structures had slight pulmonary swelling and showed a milder inflammation. In contrast, some severe lesions appeared in lung tissue for the pcDNA3.1(+) vector group and PBS group, including alveolar septa and interstitium thickening, alveolar congestion, and a large amount of inflammatory cell infiltration, as noted under light microscope observation (Figure 5A). Furthermore, immunizations with pcDNA3.1(+)/CPSIT_p7 vaccine and CPSIT_p7 protein vaccine reduced the *C. psittaci* load in the lung compared with the negative control and blank group (Figure 5B).

### 2.6. pcDNA3.1(+)/CPSIT_p7 Vaccine Suppressed C. psittaci Dissemination in BALB/c Mice

**Figure 6:**
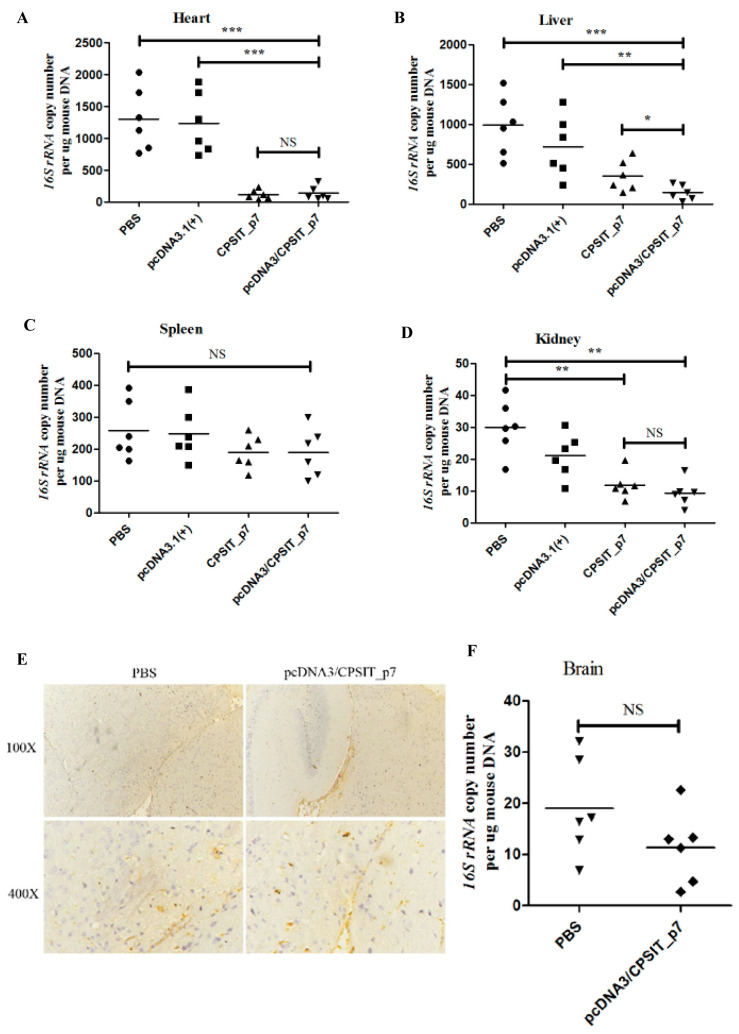
Detection of *C. psittaci* load in various organs of infected mice. Quantitative real-time PCR was used to measure the *16s rRNA* DNA concentrations in the (**A**) heart, (**B**) liver, (**C**) spleen, (**D**) kidney, and (**F**) brain of mice in each group. The results were normalized to the mice’s DNA concentrations using the Mann–Whitney test. The points correspond to three samples that were separately extracted from each mouse. Horizontal lines represent mean values (* *p* < 0.05, ** *p* < 0.01, *** *p* < 0.001, NS = no significance). (**E**) Illustrative immunohistochemical photographs of the brain from different groups of infected mice after immunization.

**Figure 7:**
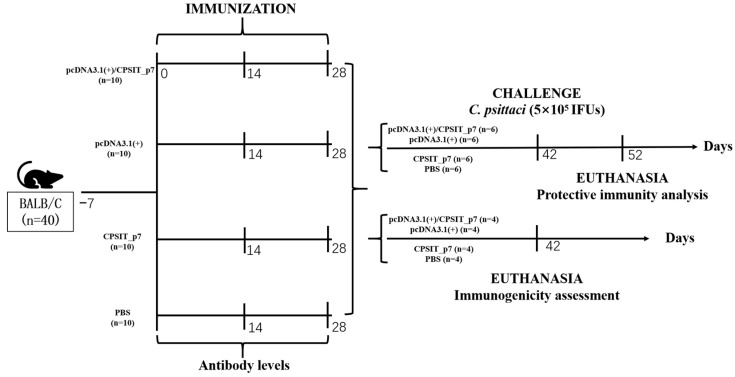
The timeline of immunization and overall in vivo work through the terminal point on day 52.

To better understand the *C. psittaci* dissemination in infected mice after vaccination, we detected the *C. psittaci* load in infected mice’s heart, liver, spleen, kidney and brain tissues by RT-PCR. We found that the load in the heart, liver, and kidney of mice in pcDNA3.1(+)/CPSIT_p7 plasmid group and CPSIT_p7 protein group was significantly reduced compared with that of pcDNA3.1(+) vector group and PBS group (Figure 6A,B,D). Significantly, the pcDNA3.1(+)/CPSIT_p7 plasmid group had a lower load in the liver than the CPSIT_p7 protein group (Figure 7B). There was no significant difference in the load in the spleen in all groups (Figure 6C). The results of PCR and immunohistochemical assays are the same, that the load in the brain of mice in pcDNA3.1(+)/CPSIT_p7 plasmid group and PBS group were both low (Figure 6E,F). In addition, the latter had a higher load than the former, but there was no statistical significance between them (Figure 6F).

## 3. Discussion

Vaccine research has been a very effective way to prevent and treat *Chlamydia* infection and transmission. A DNA vaccine is in line with the development trend of chlamydial vaccine research because of its safety, convenience of operation, and the unique advantages of mimicking the expression form of antigen in vivo [6,7]. This study constructed the *C. psittaci* plasmid protein eukaryotic expression vector pcDNA3.1(+)/CPSIT_p7, which could be stably expressed in eukaryotic cells. Intramuscular injection of mice induced high levels of plasmid protein-specific antibodies. Anti-infection protection experiments showed that pcDNA3.1(+)/CPSIT_p7 could effectively accelerate the clearance of *Chlamydia* in the infection site, reduce inflammatory factors, and inhibit the spread of *C. psittaci* to the distal tissues and organs. These results indicated that pcDNA3.1(+)/CPSIT_p7 could produce anti-infection immune protection.

The autoimmunity characteristics of plasmid protein Pgp3 and its role in chlamydial pathogenesis determine that a protein-based DNA vaccine can also be used as a candidate antigen for vaccine research against chlamydial infection [23,24,25]. We built pcDNA3.1(+)/CPSIT_p7. A KOZAK sequence, GCCACC, was inserted after the upstream restriction enzyme *BamHI* cutting site to enhance eukaryotic vector expression. After multiple immunizations, eukaryotic vectors could induce specific antibody production, but the antibody level was far lower than that of subunit antigen CPSIT_p7. Although the KOZAK sequence was inserted, the immunogenicity and immune effect differed. Although the induced antibody levels differed, subsequent anti-infection experiments showed diametrically opposite effects. pcDNA3.1(+)/CPSIT_p7 can induce anti-infection immune protection comparable to subunit antigen, which can effectively accelerate the clearance of *Chlamydia* in the lungs and inhibit the spread of *C. psittaci* to distal tissues and organs. Although the level of specific antibodies induced by a eukaryotic vector is low, it may contain many protective antibodies. In addition, Freund’s adjuvant was used to enhance the immunogenicity of the protein antigen CPSIT_p7, which would also greatly enhance the immunogenicity and immune effect of the protein antigen, which is consistent with previous research results [12,13]. However, we still need to study how to increase the immunogenicity and effect of DNA vaccines in subsequent experiments, including improving the expression of target genes by plasmids, enhancing the delivery effect of plasmids, and using adjuvants [6]. We are currently conducting an experiment synthesizing the CpG ODN1826 base sequence as an adjuvant; exogenous mixed with eukaryotic vector, given to immunize mice, yielded good results.

Unfortunately, we did not study the cellular immune response induced by pcDNA3.1(+)/CPSIT_p7 immunization. Detection of a eukaryotic vector by antibody level can induce a strong humoral immune response. We isolated mouse spleens for periodic analysis of cellular immune responses. We speculated that spleen cells were in a non-growth state due to the long treatment time of the red blood cell lysis buffer. Liang et al. also failed to evaluate the cellular immune response induced by protein antigens [26]. However, previous studies have demonstrated that restricted CD4^+^ T cell primary histocompatibility complex class II (MHC-II) is required for protective immunity by vaccines, which enhance clearance of chlamydial infection by producing IFN-γ [27,28]. After vaccination, some circulating Th1 lymphocytes become mucosal tissue-resident memory cells that rapidly reduce the chlamydial load upon reinfection [27]. For optimal protection, chlamydial vaccines must induce Th1-cytokine-biased CD4^+^T cells, especially IFN-γ, and humoral responses [17,29,30]. Our previous studies also found that both subunit antigens and polypeptide antigens could induce the secretion of Th1-biased cytokines and produce protective effects [19,31]. In addition, we also detected significant differences in the reduced levels of IFN-γ in lung tissues after *C. psittaci* infection. We hypothesized that the eukaryotic vector pcDNA3/CPSIT_p7 might induce a Th1-biased cellular immune response, which needs further study.

Evaluating the lung tissues of mice infected with *C. psittaci* after immunization can effectively accelerate the clearance of *C. psittaci* at the injection site. The subunit antigen CPSIT_p7 has been shown to have a protective effect against disease due to the excellent immunogenicity and the combination of adjuvants [19]. We have previously demonstrated that pcDNA3.1(+)/CPSIT_p7 produces immune potency comparable to subunit antigen, with reduced chlamydial load at the site of infection, milder lesions, and lower levels of inflammatory factors. However, the levels of *Chlamydia* and IFN-γ in lung tissues differed from those in previous studies. We speculate that there are two possible reasons: first, the different number of *C. psittaci* passages and the different genetic and cultural backgrounds of each batch of infected mice caused these differences; second, these differences are caused by the characteristics of the immune antigen itself, the specificity of the DNA antigen and the subunit antigen itself, which may be expressed in different forms in vivo. It has been suggested that plasmid protein antibodies provide only partial protection during *C. psittaci* infection [19,32], and our results are consistent with that notion.

*C. psittaci* is a highly infectious and pathogenic pathogen that can be transmitted from sick birds to humans through the respiratory tract. Invading distal organs or tissues through blood, *C. psittaci* infection can cause systemic reactions, including lung, heart, liver, spleen, lung, kidney, heart, and other organs and tissues, resulting in systemic diseases such as pneumonia, myocarditis, hepatitis, nephritis, and even encephalitis and death if not treated in time [33]. Therefore, we further evaluated whether DNA vaccines could inhibit the ability of *C. psittaci* to spread to multiple tissues and organs. Pgp3 is a virulence factor, and its related DNA vaccine also plays an important role [20]. Our study found that pcDNA3.1(+)/CPSIT_p7 could ameliorate the degree of *C. psittaci*-induced lesions. It can reduce the load of *C. psittaci* in each tissue, inhibit the spread of *C. psittaci* to the distal organs, and effectively strengthen the clearance of *C. psittaci*. Our previous study also showed that *C. psittaci* infection in mice caused symptoms of chlamydial infection in various tissues and organs throughout the body, especially in the lungs. Donati [20] and Li et al. [21] also found that the DNA vaccine targeting gene *Pgp3* could inhibit the ascending infection of *C. trachomatis* after immunizing mice, significantly reducing the chlamydial load in vaccinated mice and accelerating the clearance of infection. Our study found the same result. It is noteworthy that the spleen, an important peripheral immune organ, plays a pivotal role in the host immune response against pathogen infections. There are a variety of immune cells conducive to the capture and control of pathogens in the spleen, especially the macrophages [34,35,36]. If intracellular elimination fails, *Chlamydia* may survive in the macrophages. Thus, *Chlamydia* may be disseminated from the infection site to distant viscera or sites via blood and lymphatic circulation in macrophages, which are regarded as the delivery carriers [37,38,39]. However, there are anatomical barriers to infection in the central nervous system, such as the blood–brain barrier, that can restrict the entry of pathogens into the brain [40]. It is inferred that the lower infection load in brain tissues is because of the blood–brain barrier. Therefore, the infection load is lesser in the spleen and brain as compared to other tissues.

In addition, *C. psittaci* infection can cause encephalitis [1]. Our study on the behavior of infected mice found that some individuals in the control group showed manic phenomena later in the infection. Their activity was very heightened, sometimes biting themselves and their partners. However, none of the pcDNA3.1(+)/CPSIT_p7 mice showed any related phenomenon. The difference in *C. psittaci* load in the brain tissue might cause the difference in this behavior. Therefore, we tentatively studied the brain tissue of mice in the PBS group and the pcDNA3.1(+)/CPSIT_p7 group. Immunohistochemical results showed a small amount of *C. psittaci* load in the brain tissues of each group. The results of RT-PCR showed that the *C. psittaci* load in the brain tissue of mice immunized with PBS might be higher, but the statistical difference was not significant. We speculate that it is challenging for *C. psittaci* to cross the blood–brain barrier, and *Chlamydia* has been largely cleared by the time *C. psittaci* spreads to the brain tissue. In addition, short-range infection of *C. psittaci* may also limit diffusion. The inhibitory effect of pcDNA3.1(+)/CPSIT_p7 on *C. psittaci* diffusion still needs further evaluation.

Our previous studies also found that both subunit vaccine with antigens or polypeptide antigens based on the CPSIT_p7 protein could induce good immune protection against chlamydial infection. However, there are challenges in numerous expressions and purification of target protein-related subunit vaccines. In our study, we used the pcDNA3.1(+)/CPSIT_p7 DNA vaccine as the experimental group and the recombinant CPSIT_p7 protein subunit vaccine as the positive control group. Some results had shown that these two vaccines induced similar immuno-protection against *C. psittaci* infection. Although this DNA vaccine has huge potential, it still needs to overcome a series of limitations and challenges. Low immunogenicity is a potential disadvantage; however, multiple types of adjuvants including cytokines, the granulocyte-macrophage colony-stimulating factor, the CpG oligodeoxynucleotide, etc., have been proven helpful for enhancing the immunogenicity of DNA vaccines [41,42,43]. Compared with viral vectors, DNA vaccine delivery systems are safer, but the risk of insertional mutagenesis stills exists [44,45,46]. So far, it is difficult to evaluate the safety of the DNA vaccine by determining the risk of insertional mutagenesis accurately. Optimizing gene detection instrumentation and techniques, such as qPCR, qRT-PCR, or fluorescence-based assays, would probably be highly useful for the safety assessment of DNA vaccines [47]. Further studies on the safety of DNA vaccines are essential and critical before they can be used in humans.

In conclusion, evaluation of the protective immunity of pcDNA3.1(+)/CPSIT_p7 vaccination in mice demonstrates that that pcDNA3.1(+)/CPSIT_p7 DNA vaccine has good immunogenicity and immunity protection effectiveness against *C. psittaci* infection in BALB/c mice, and could suppress *C. psittaci* dissemination. Our study paves a promising way for the development of an effective anti-*Chlamydia* DNA vaccine and provides for the development of DNA vaccines against chlamydial infection and other intracellular pathogens.

## 4. Materials and Methods

### 4.1. Construction and Identification of the pcDNA3.1(+)/CPSIT_p7 Plasmid

According to *the CPSIT_p7* gene reported by the GenBank database (No. CP002550.1), we designed the specific forward primer (5′-CGCGGATCCATGGGTAATTCTGGTTTTTAC-3′) and reversed primer (5′-CCGCTCGAGTTAACCATTTGTTTGTTGTTTT-3′). The full-length *CPSIT_p7* gene was PCR-amplified from *C. psittaci* 6BC genomic DNA (NCBI Reference Sequence: NC_017288) and inserted into the pcDNA3.1(+) plasmid with *BamHI* and *XhoI* restriction sites. After verification by PCR amplification, agarose gel electrophoresis, and DNA sequencing, recombinant plasmid pcDNA3.1(+)/CPSIT_p7 was transformed into *E. coli* BL21 (DE3). *CPSIT_p7* gene replicated in *E. coli* BL21 was extracted and purified using the Endo-Free Plasmid Maxi Kit (Omega, Norcross, GA, USA), and then stored at 4 °C before use.

### 4.2. Transfection

HeLa cells were seeded at a density of 1 × 10^6^ cells/well on 6-well culture plates with DMEM supplemented with 10% fetal bovine serum (Gibco, Grand Island, NY, USA), and cultured at 37 °C in the presence of 5% CO_2_ until they reached 80–90% confluence. Transfection steps were carried out according to Lipofectamine^®^ 2000 reagent (Invitrogen, Carlsbad, CA, USA) instructions. Briefly, the mixed lipofectamine 3000/pcDNA3.1(+)/CPSIT_p7 and lipofectamine 3000/pcDNA3.1(+) at a volume/mass ratio of 10 μL/5 μg was added into corresponding wells. The empty pcDNA3.1(+) vector was the negative control, and PBS served as a blank control. The protein expression was detected 48 h after transfection.

### 4.3. Western Blot Analysis

After 48 h, the HeLa cells transfected with pcDNA3.1(+)/CPSIT_p7, empty pcDNA3.1(+) vector, and PBS were identified by Western blot analysis. Protein samples were taken for 12% SDS-PAGE and transferred to polyvinylidene fluoride (PVDF) membranes. The membranes were soaked in 5% skim milk solution for 2 h at 37 °C, and then incubated with 1:500 dilutions of rabbit anti-*C. psittaci* sera (a gift from Zhong G, the University of Texas Health Science Center at San Antonio, TX, USA) at 4 °C overnight. Washed membranes were incubated with HRP-conjugated goat anti-rabbit IgG (Abcam, MA, USA) with 1:2000 dilutions for 1 h at 37 °C. Proteins were visualized using the Omni-ECL™Pico Light Chemiluminescence Kit (Epizyme Biotech, Shanghai, China).

### 4.4. Mice Care and Immunization

All immunization and studies were performed on six-week-old special pathogen-free BALB/c female mice (Hunan SJA Laboratory Animal Co., Ltd., Changsha, China). The animals were reared in specific pathogen-free conditions at 26 °C, fed with sterile food and water once daily, and accommodated for a week before the experiments. Forty mice were split into four groups (ten mice per group) at random: pcDNA3.1(+)/CPSIT_p7 eukaryotic expression plasmid group (experimental), empty pcDNA3.1(+) vector group (negative control), the recombinant CPSIT_p7 protein group (positive control), and PBS group (blank control). All groups were immunized three times, with two weeks between immunizations. Each mouse in the experimental group and negative control group was given an intramuscular injection of 100 μg pcDNA3.1(+)/CPSIT_p7 plasmid and empty pcDNA3.1(+) vector (diluted to 100 μL with sterile PBS solution), respectively. In the positive control group, each mouse was administered 30 μg CPSIT_p7 protein emulsified in an equivalent volume of FA (complete for the first injection and incomplete for the other 2 times) in a volume of 100 μL. At the same time point, the blank control groups received 100 μL PBS solution. Blood was collected by tail bleeding before each immunization. Blood samples were stored overnight (4 °C) and the serum was isolated later through centrifugation (4000× *g*, 20 min, 4 °C). The serum was prepared and stored at −80 °C. Two weeks after the final immunization, four mice per group were euthanized for the evaluation of the humoral immunity and cellular immunity induced by the vaccines, then the remaining mice were intranasally infected with 5 × 10^5^ IFU of *C. psittaci* and sacrificed at day 10 postinfection, in order to evaluate the immune protective efficacy of the vaccines (Figure 7).

### 4.5. ELISA Analysis of Antibody Levels

To evaluate the vaccine-induced humoral response, the levels of anti-mouse antigen-specific serum IgG were detected by enzyme-linked immunosorbent assay (ELISA). Briefly, 96-well ELISA plates were coated with recombinant protein CPSIT_p7 (1 μg/well) in bicarbonate buffer (0.05 mol/L, pH 9.6) and incubated overnight at 4 °C. After washing with PBS solution containing 0.05% Tween 20 (PBST), the plates were blocked with blocking buffer (PBST containing 5% skimmed milk, 200 μL/well) at 37 °C for 2 h. Then, the serum samples, as mentioned above, were diluted at 1:200 in blocking buffer, and doubling dilution was made serially. After incubation at 37 °C for 2 h and washing, 100 μL horseradish peroxidase (HRP)-conjugated goat anti-mouse IgG was diluted at 1:10,000 in blocking buffer to each well for 1 h at 37 °C. After washing, each well was incubated with 100 μL 3, 3′, 5, 5′-tetramethylbenzidine substrate at 37 °C for 15 min, then the same volume of 2 M H_2_SO_4_ was added to stop the color reaction. Finally, the absorbance was measured at 450 nm.

### 4.6. Preparations of C. psittaci and Intranasal Challenge

*C. psittaci* strain 6BC (ATCC VR-125) was propagated by HeLa 229 cell (ATCC CCL-2.1) culture, as mentioned previously. Elementary bodies (EBs) were harvested by density-gradient centrifugation and stored at −80 °C. Then, EBs were counted by indirect immunofluorescence assay (IFA), and the EB titer was represented as inclusion-forming units/mL (IFUs/mL). In order to evaluate the immune protective efficacy of the vaccine, immunized mice of each group were intranasally infected with 5 × 10^5^ IFU of *C. psittaci* two weeks after the final immunization (Figure 1).

### 4.7. Indirect Immunofluorescence Assay

HeLa 229 cells were grown on 24-well culture plates and infected with the supernatants of lung homogenates. At 48 h after infection, cells were fixed in 4% paraformaldehyde, permeabilized in 0.3% TritonX-100, and stained with primary and secondary antibodies to visualize *C. psittaci*. Inclusion was observed by fluorescence microscopy assay, and the number of inclusions was counted based on the area and dilutions and expressed as IFUs.

### 4.8. Detection of Cytokine Levels in Lung

Lung tissue homogenates from *C. psittaci*-infected mice in each group were prepared and centrifuged at 6000 rpm for 15 min at 4 °C. The production of cytokines IL-6 and IFN-γ was determined using commercially available ELISA kits (eBioscience, San Diego, CA, USA), separately, following the instructions provided by the manufacturer.

### 4.9. Histopathology

Lung tissues isolated from mice after euthanasia were fixed with 4% paraformaldehyde, embedded in paraffin, serially sectioned, and stained with hematoxylin–eosin (H&E). In order to detect *C. psittaci*, an UltraSensitive™SP (Rabbit) IHC Kit (Maixin, Fuzhou, China) was used for streptavidin–peroxidase (S–P) immunohistochemistry (IHC). The results were observed by light microscopy.

### 4.10. Quantitative PCR

DNA was extracted from the heart, liver, spleen, kidney, and brain tissues using TIANamp Genomic DNA Kit (TIANGEN BIOTECH, Beijing, China), and as a template for the quantitative PCR amplification of *C. psittaci 16S rRNA* sequence and mouse *β-actin* sequence. The primers for *C. psittaci 16S rRNA* sequence amplification were: forward, 5′-TCCGCAAGGACAGATACACA-3′ and reverse, 5′-ACCCAGGCAGTCTCGTTAGA-3′. Forward primer for mouse *β-actin* sequence amplification was 5′-CCTTCCTTCTTGGGTATGGA-3′, and the reverse was 5′-ACGGATGTCAACGTCACACT-3′.

### 4.11. Animal Ethics Statement

Six-week-old special pathogen-free BALB/c female mice were obtained from Hunan SJA Laboratory Animal Co. Ltd. (Changsha, China, Animal Production License No. SYXK 2021-0002) and kept in the Animal Experiment Research Center at the University of South China. All experimental protocols involving animals were approved by the Ethics Committee of the Animal Experiment Research Center at the University of South China. The mice operations conformed to National Laboratory Animal Care and Use Guidelines.

### 4.12. Statistical Analysis

Statistical differences between groups for *C. psittaci* load, antibody titers, and cytokine levels were analyzed using one-way analysis of variance (ANOVA) with the Student–Newman–Keuls test. Results were considered statistically significant at *p* < 0.05, and all statistical analyses were performed with the software package SPSS 18.0.

## Data Availability

The data used to support the findings of this study are included in the article.
